# Retraction: On-farm hydro and nutri-priming increases yield of rainfed pearl millet through physio-biochemical adjustments and anti-oxidative defense mechanism

**DOI:** 10.1371/journal.pone.0272535

**Published:** 2022-08-17

**Authors:** The PLOS ONE Editors

The *PLOS ONE* Editors retract this article [1] because it was identified as one of a series of submissions for which we have concerns about authorship, competing interests, and peer review. We regret that the issues were not addressed prior to the article’s publication.

NKGupta, RKS, TJ, HMA, RK, and MHS did not agree with the retraction. SG, JS, NKGarg, DS, and AAAH either did not respond directly or could not be reached.
